# A scoping review on the body awareness rehabilitation after stroke: are we aware of what we are unaware?

**DOI:** 10.3389/fneur.2024.1497052

**Published:** 2025-01-07

**Authors:** Davide Cardile, Viviana Lo Buono, Francesco Corallo, Angelo Quartarone, Rocco Salvatore Calabrò

**Affiliations:** IRCCS Centro Neurolesi Bonino-Pulejo, Messina, Italy

**Keywords:** chronic stroke, subacute stroke, neuropsychological assessment, body awareness, neurorehabilitation

## Abstract

Body awareness (BA) is a complex multi-dimensional construct that refers to the subject’s ability to consciously perceive and integrate sensory and proprioceptive information related to the position, movement, and balance of one’s own body and body parts. Since it involves multiple brain regions and include different functional networks, it is very often affected by cerebrovascular damage such as stroke. Deficits in the ability to monitor our actions and predict their consequences or recognize our body parts and distinguish them from those of others may emerge after stroke. In this study, we decided to explore whether specific treatments targeting BA are discussed in current literature, and whether BA is considered as an outcome in neurorehabilitation processes for stroke patients. To achieve our goal, a scoping review on this often-underreported problem was performed. After analyzing the existing literature, emerged BA in stroke patients is rarely assessed or rehabilitated through specific stimulation or rehabilitation protocol. Additionally, treatment outcomes related to BA are often considered only from a “physical” perspective such as improvements in walking, balance, or the movement of specific body parts, rather than from a proprioceptive standpoint. Further research is needed to facilitate developing early and effective intervention strategies for the recovery of BA after stroke.

## Introduction

A stroke is characterized by a sudden, focal, nonconvulsive neurological deficit of vascular origin, which can be either ischemic or hemorrhagic (1). This cerebrovascular event often leads to significant disabilities, with motor impairments being one of the most prevalent, affecting approximately 80% of stroke survivors (2). Beyond motor dysfunction, individuals may also experience a range of other deficits, including cognitive alterations, sensory disruptions, spasticity (3, 4), hemiparesis, diminished postural stability, and impairments in gait and balance (5, 6). Given the nature of these post-stroke impairments, it is highly probable that body awareness (BA) is affected in stroke survivors. The term BA refers to an individual’s ability to consciously perceive and integrate sensory and proprioceptive information related to their own body. This includes not only the perception of body position, movement, and balance but also the sense of ownership over one’s body parts, often referred to as “embodiment” (7).

BA is a multi-dimensional construct that involves a complex system that includes multiple brain regions and functional networks, notably involving the thalamus, insula, and cerebellum (8, 9). The complexity of the construct derives not only from the involvement of different anatomical sites, but also from the interaction of various sensory modalities (the somatosensory, vestibular, and visual systems) along with higher-order cognitive processes that interpret and modulate these sensory inputs (10). Not surprisingly among the most commonly used assessment instruments there are multidimensional tests like the Body Awareness Questionnaire (BAQ), the Body Perception Questionnaire (BPQ), and the Multidimensional Assessment of Interoceptive Awareness (MAIA), which assess motor awareness/perception, and the level of consciousness related to one’s internal sensations (11–13).

The cognitive, motor, and emotional abnormalities that manifest after a stroke can vary depending on the hemisphere and on the site that is damaged. In stroke patients often neuropsychological deficits are generally observed in the hemispace contralateral to the brain lesion, both at the perceptual and visual levels (14–16). The right hemisphere, in particular, is closely linked to emotional regulation, body image, and visuospatial skills (17, 18). One of the dysfunctions that makes these complaints most evident is unilateral spatial neglect, often referred to simply as “neglect,” in which patients fail to recognize or respond to novel or meaningful stimuli presented on the side opposite the hemispheric lesion (19). This deficit significantly impacts the patient’s attention, rendering them unable to shift attention toward the impaired visual hemifield. Consequently, this condition results in an inability to attend to, move the eyes toward, or engage with the contralesional space (20, 21).

Anosognosia can also be considered as a disorder of BA. This neurological condition is characterized by a patient’s lack of awareness or denial of their own illness or deficits and is often interpreted as a profound disruption in BA (22). Specifically, anosognosia for hemiplegia, where patients are unaware of paralysis on one side of the body following a stroke, exemplifies how this condition is fundamentally tied to a breakdown in the brain’s ability to integrate and process sensory, motor, and cognitive inputs that contribute to BA (23). Patients with right hemispheric strokes, particularly those affecting the inferior parietal lobule and insula, are more likely to exhibit anosognosia for hemiplegia, suggesting that these regions are central to maintaining an accurate, conscious representation of the body (24, 25). The inferior parietal lobule, in particular, is critical for integrating sensory input with motor commands, which allows individuals to perceive their own actions and bodily states (26). When this integration fails, as in the case of anosognosia, patients may lose the ability to perceive or acknowledge deficits in their motor or sensory functions.

A related condition called somatoparaphrenia, patients with anosognosia deny ownership of their own paralyzed limbs, further illustrates how anosognosia represents a breakdown in the brain’s systems for BA (27, 28). Somatoparaphrenia involves more than just a lack of awareness of motor impairment; patients often perceive their affected limbs as belonging to someone else or as foreign objects entirely. This distortion of body ownership highlights the degree to which anosognosia disrupts the brain’s ability to maintain a coherent, integrated sense of the body (25). The right temporoparietal junction, another region linked to body ownership and the sense of self, is often implicated in these disturbances, underscoring its role in maintaining BA (29, 30).

Roth (31) found that in addition to proprioception, even body image disorders are particularly associated with damage to the right parietal lobe. Even left hemisphere damage may affect body awareness, since motor planning and execution deficits typical of apraxic patients, for example, negatively affect BA as they may have difficulty in monitoring and understanding the correct use of their extremities in space (32, 33). Left hemispheric damage may also be associated with a decreased awareness of voluntary movements of one’s body, often in association with damage to motor and premotor areas (34). Damages in these areas can impair motor awareness and internal representation of movements, affecting the ability to imagine or remember bodily movements, which may contribute to a distortion of body image and perception (35). Body image refers to the visual representation of the body, which is formed through the graphic depiction of ourselves and results from the integration of multisensory neural inputs. Body image is fundamentally a visual representation of the self, shaped through the integration of multisensory neural inputs. This concept involves affective and memory-related contributions from the limbic system, while its semantic and lexical aspects require input from the language and spatial processing areas of the parietal lobes in both hemispheres (8).

All the syndromes we have discussed illustrate how localized brain damage can disrupt the integrated representation of the body, leading to profound alterations in self-perception and even a significant number of stroke survivors experience significant disruptions with brain’s representation of the body (36). Even if these neuropsychological conditions are not necessarily permanent, when symptoms and syndromes such as those described above occur, they profoundly affect the individual’s ability to perform daily activities (37). The limitations imposed by these conditions often lead to a partial or total dependence on others for assistance, which, compounded by the sudden and unfamiliar nature of the acquired condition, contributes to the development of anxiety and depressive symptoms in the patient (38). BA is a fundamental component self-awareness, manifesting through one’s interactions with the environment and the external world (39). Since BA disorders are relatively common and are associated with both psychiatric and neurological conditions there is a growing interest in therapeutic interventions aimed at enhancing this awareness (40–42).

In order to gain a deeper understanding of this complex issue, we conducted a scoping review of the existing literature. The aim of this study was to explore whether specific treatments targeting BA are discussed in the literature, and whether BA is considered as an outcome in neurorehabilitation processes for stroke patients.

## Methods

**Figure 1:**
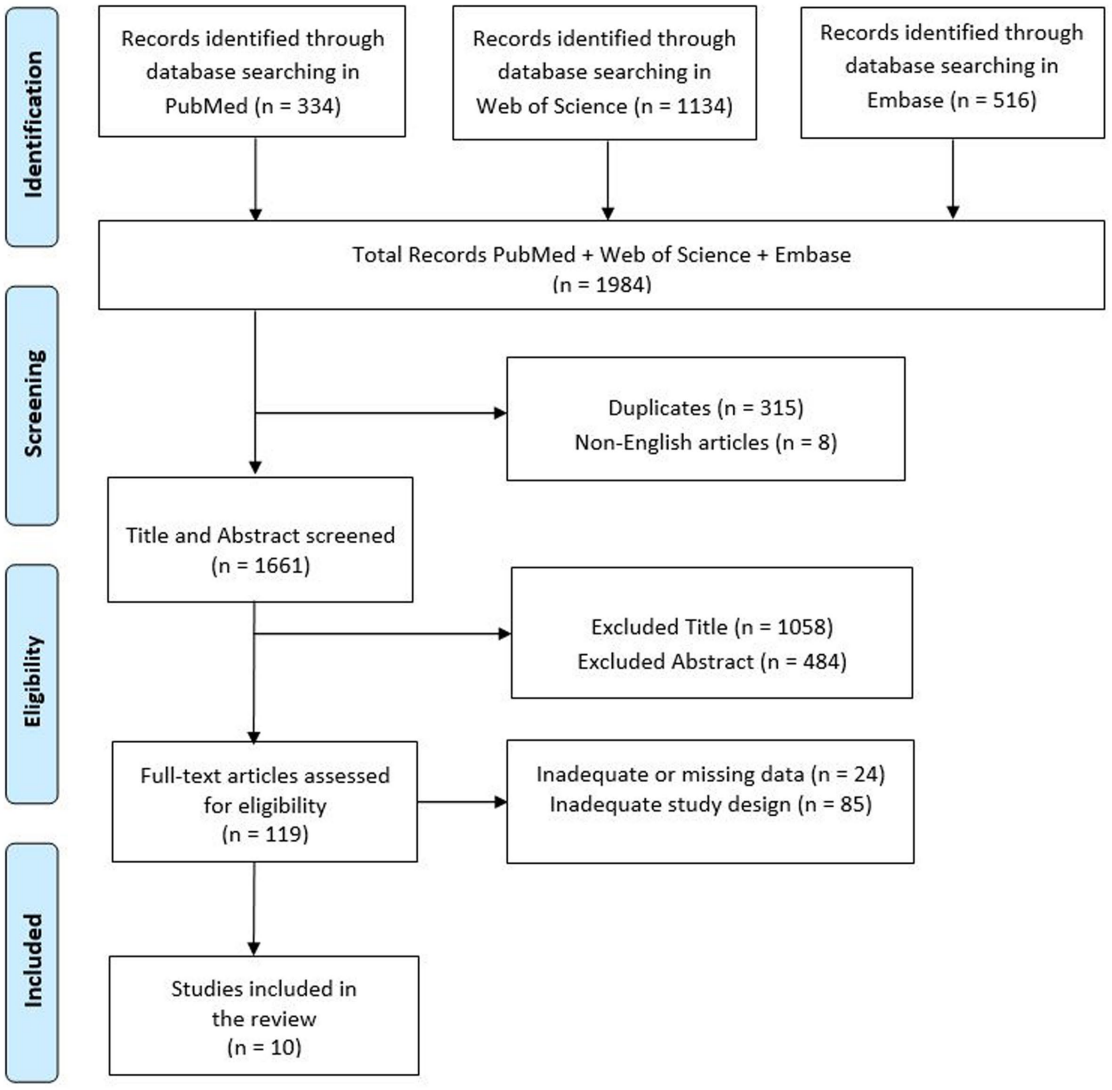
Search and selection of eligible articles.

A review of currently published studies was performed on 16.04.2024 in the following databases: PubMed, Web of Science and Embase. The search was carried out using the following search string: ((chronic stroke) OR (subacute stroke)) AND (rehabilitation) OR (psychological intervention) AND (body awareness). Initially all articles were reviewed based on titles and abstracts by two investigators (D.C., V.L.B.), who independently collected data to minimize the risk of bias (e.g., publication bias, delay bias, language bias). Full-text articles deemed suitable for the study were then read by these researchers, and in case of disagreements regarding inclusion or exclusion criteria, a final decision was made by a third researcher (R.S.C.). PRISMA diagram was added to describe the sequence of steps (identification, screening, eligibility, and inclusion) for the collection and determination of qualified studies (43) as shown in Figure 1. Disagreements between reviewers were resolved by consensus. Finally, this study was registered on Open Science Framework (OSF) (44) at: doi: 10.17605/OSF.IO/NVGH4.

### PICO evaluation

We defined our combination of search terms using a PICO (population, intervention, comparison, outcome) model (45). The population was limited to patients with subacute or chronic stroke. The intervention included all studies who investigate patients’ BA implementing a rehabilitation program. The comparison was evaluated considering the rehabilitative interventions that produced BA changes in chronic or subacute stroke patients. The results included any improvements in clinical, psychological and motor aspects during the rehabilitation process.

### Inclusion criteria

A study was included if it described or investigated typology and effects of neurorehabilitation treatment in subacute or chronic stroke patients. Only studies conducted on human populations and published in English or Italian were considered for this review. Original or protocol studies of any type that presented canonic or off-label neurorehabilitative BA treatment and its influence on stroke patients were taken into consideration for this paper.

### Exclusion criteria

A study was excluded if there was a lack of BA treatment models or if it involved animal and/or cellular models. Systematic, narrative, or integrative reviews were also excluded, although their reference lists were checked and included if appropriate. All articles written in languages other than Italian or English were excluded. No restriction due to the year of publication was adopted.

### Quality assessment of included studies—risk of bias

The Newcastle-Ottawa Scale (NOS) was used to assess each study, following the criteria of the Cochrane Non-Randomized Studies Methods Working Group. NOS is an easy and convenient tool used to assess the methodological quality of non-randomized interventional studies. Evaluation includes key areas such as subjects’ selection, comparability of groups, and evaluation of outcomes. Each study can be awarded with a maximum of one point (also defined as “star”) for each numbered item of the scale. A maximum of four point can be assigned for “Selection” while, respectively, three and two points can be assigned for “Outcome” and “Comparability” sections. Higher scores correspond to higher quality for each section. NOS allows for a systematic assessment of potential bias, offering insights into the strengths and limitations of the reviewed studies (Table 1).

**Table 1:** Newcastle-Ottawa Scale results for each study involved in this review.

Study	Selection	Comparability	Outcome assessment	Total score
Tambone et al. 2021 (46)	3	0	2	5
Gomez-Andres et al. 2020 (47)	2	0	2	4
Sengar et al. 2019 (48)	3	1	2	6
Battesha et al. 2022 (53)	3	2	2	7
Gandolla et al. 2015 (49)	2	2	2	6
Palsdottir et al. 2020 (54)	4	2	2	8
Varalta et al. 2019 (50)	3	2	1	6
Chiaramonte et al. 2024 (51)	2	2	2	6
Markovic et al. 2024 (55)	3	2	3	8
Sanchez-Cuesta et al. 2024 (52)	2	1	3	6

## Search results

The initial electronic data search yielded a total of 1984 potentially relevant studies on PubMed, Web of Science and Embase. Of these, 315 were duplicated and 8 were non-English articles. A total of 1,542 articles were excluded due to title or abstract (Figure 1).

Of the resulting 419 articles, 10 fully met the inclusion criteria and were therefore included in the review. All articles delve into different types of treatment. Features of studies included, such as aim and design of the study, patients’ lesion/symptomatology, intervention type and clinical outcomes were summarized in Table 2. A summary of these studies is shown in Table 2 that shows the type, purpose and population of study, the lesion site and concurrent symptoms, the type of intervention, the outcome measures and the results.

**Table 2:** Summary of studies included in the research.

Authors	Study type	Population	Lesion site	Symptomatology	Aim	Methods/Intervention type	Outcome measures	Results
Tambone et al. 2021 (46)	Nonrandomized Non-controlled Trial	12 patients with ischemic (*n* = 8) or hemorrhagic (*n* = 4) stroke.	Fronto-temporo-parietal left-hemisphere damage.	Chronic nonfluent aphasia, balance, gait, and mobility deficits. No comprehension deficits.	Investigate if body ownership can promote motor recovery in stroke patients.	3 weekly sessions of virtual reality training were performed for 11 weeks. Through the different perspective used (1st or 3rd person) patients could experience or not experience the illusory body control of a virtual avatar	DGI, TUG, 10-Meter WT, WGS, WWT, BBI, BBS_I_, 30 Seconds SST, RMI.	Only patients who experienced the first-person perspective improved gait and balance.
Gomez-Andres et al. 2020 (47)	Pilot study	22 chronic stroke (mean: 1 year and 10 months from the onset) patients with lower extremity paresis	Mainly located at the subcortical level and in the brainstem.	Slight to moderate paresis of lower extremity. No major cognitive impairments and no psychiatric or neurological comorbidity.	To evaluate the impact of auditory stimulation in improving gait in chronic stroke patients.	Alterations in frequency spectra of footstep sounds while walking were induced via a system that selectively amplifies and equalizes the signal in order to produce distorted auditory feedback.	Body feeling questionnaire [felt speed (slow versus quick)], body weight (light versus heavy), body strength (weak versus strong), and body straightness (stooped/hunched versus straight, agency of the walking sounds they heard, the vividness of the bodily experience, surprise about the bodily feelings, and feet localization), self-assessment manikin [Emotional valence, arousal, and dominance], BI.	The amplification of natural walking sound or the augmenting of low-frequency bands reduce asymmetry index of stance and stride times. High-frequency band instead led to opposite results.
Sengar et al. 2019 (48)	Clinical Trial	30 patients with chronic stroke from with an age between 45 and 65 years old.	Middle cerebral artery chronic stroke.	Right (*n* = 13) or left (*n* = 17) paretic limb. No acustic, visual or vestibular impairment. No aphasia.	To compare dual-task training efficacy using 2 instructional sets to improve gait parameters in chronic stroke patients.	Patients were divided into two groups: the first received dual-task training with fixed priority instructional sets, while the second received variable priority instructional sets.	MMSE, 10 min WT, E-FAP, TUG.	Gait parameters improved in both groups. Group 2 showed better results.
Battesha et al. 2022 (53)	Randomized Controlled Trial	30 chronic stroke patients with an age between 45 and 65 years old.	Middle cerebral artery stroke leading to right cerebral hemisphere infaction.	Left hemiplegia, impairment of posture stability. All patients can stand and walk even if using an assistive device.	To evaluate the effect of maze control training on kinesthetic awareness in a chronic stroke population.	Patients were divided into two groups: group A was treated with conventional physiotherapy, while group B received the same treatment of group A in addition to maze control training.	Sway index, MRC, BBS_II_, BID.	Sway index and risk of fall decreased in both groups. Knee proprioception improves only group B.
Gandolla et al. 2015 (49)	Semi-Randomized Controlled Trial	14 chronic stroke patients (age 44 ± 14 years)	Not provided.	Weakness of tibialis anterior muscle. No significant language, cognitive or walk deficits. No high ankle spasticity.	To describe neural correlates in chronic stroke patients receiving functional electrical stimulation (FES) for foot-drop correction.	During a continuous 10 min scanning session patients perform ankle dorsal-plantar flexion (volitional and passive) and receive FES (present and absent). fMRI was used to examine neural correlates under these conditions.	Davis gait evaluation Protocol, MRC scale, 6 min WT, TAAI, Capacity Score.	The network shared by patients was bilateral sensorimotor and supplementary motor activations. Moreover, patients who experienced the carryover effect has responses comparable to control groups
Palsdottir et al. 2020 (54)	Randomized Controlled Trial	101 stroke patients (73 sub-acute, 28 chronic) between 47 and 80 years old. Stroke was ischaemic in 89 patients and hemorrhagic in 11.	Not provided.	Not specified. Preserved ADL skills. No dementia, severe aphasia or other severe comorbidities.	To assess the long-term effect of nature-based rehabilitation (in addition to standard care) on post-stroke patients.	Patients were divided into two groups: the experimental group undergoes standard care + nature-based rehabilitation, while the control group undergoes only standard care.	NIHSS, MOCA, MFS, mRS, HAD, EQ-5D.	No statistically significant difference emerged in outcome measures. Fatigue decreased below the suggested cut-off for mental fatigue only in the experimental group. Both groups were compliant with the intervention.
Varalta et al. 2019 (50)	Single Blind Randomized Controlled Trial	12 patients with hemispatial neglect consequent to ischemic/hemorrhagic stroke occurred at least 6 months earlier.	Not provided.	Hemispatial Neglect, Absence of musculoskeletal, psychiatric or visual disorders.	To examine the effect of therapeutic neck taping on kinesthetic sensibility, neck motion and visuo-spatial abilities in stroke populations with neglect.	Patients received 30 consecutive days of real or sham neck taping. Between group comparisons were performed.	SCT, AROM, LCT, CRT, CJPET.	Cervicocephalic kinesthetic sensibility resulted in improvement by neck taping.
Chiaramonte et al. 2024 (51)	Retrospective Observational study	35 patients (age 75.31 ± 8.65 years) with motor impairment ischemic stroke occurred between 3 and 12 previous weeks (subacute).	22 had lesions in the territory of the middle cerebral artery, 6 in the posterior limb of the internal capsule, 7 lacunar strokes in the capsular region.	Severity mild to moderate, spastic hemiparesis, weakness, balance issues, disability in daily activities. No cognitive impairments, with normal vigilance, cooperation, orientation, memory, attention, decision-making, and general cognitive processing capabilities	To evaluate the effectiveness of a goal oriented proprioceptive training in subacute stroke for balance, autonomy and fall risk.	3 h a day of single/dual task goal-oriented proprioceptive training for 7 days a week, organized in two sessions of 1.5 h a day. First group underwent proprioceptive exercises, while the second group integrated both motor and cognitive exercises.	NRSP, BI, TUG, BBS_I_ and Tinetti Test.	Improvements were recorded in autonomy, balance and fall risk in both groups. However, dual task group achieves more significant improvements. The treatments proved effective in improving proprioception and balance, but only partially in improving autonomy or fall risk.
Markovic et al. 2024 (55)	Randomized Clinical Trial	100 patients (mean age 49) with mild to moderate stroke (*n* = 79) or traumatic brain injury (*n* = 21).	42 and 31 patients had, respectively, left and right hemisphere lesion, while 27 had bilateral lesions. Among all, 47 patients had focal lesion, while 53 had multifocal ones. Lesion localizations were anterior (25), posterior (16), subcortical (35) or global (9)	Moderate to severe attention impairment. No aphasia, psychiatric symptoms, or neglect. Satisfactory levels of logical reasoning, memory, and fine motor functions	To describe long-term effects of two different types of rehabilitation training.	Intensive interdisciplinary rehabilitation was performed 6 h/day, 4–5 week for a total of 8–12 weeks. CRTF intervention was performed in addition of 20 h of attention training (APT or ABAT), 3–5 times/week, for 5–6 weeks. The SAG group started this treatment less than 4 months after injury while the PAG group started it between 4 and 12 months later. After 2 years a follow-up screening was performed.	EQ-5D, OGQ, WAI, Self-reported employment conditions, sick leave, and experienced cognitive limitations in work performance.	Although the differences between the two types of treatment were minimal, the SAG group performed better on ADL, work skills and return to work. Therefore, with respect to attention a sub-acute intervention is preferable to a post-acute one.
Sanchez-Cuesta et al. 2024 (52)	Exploratory crossover clinical trial	20 patients with subacute (*n* = 4) or chronic (*n* = 16) ischemic (*n* = 7)/hemorrhagic (*n* = 5) stroke.	Not provided.	Upper limb motor impairment, No read/write alterations, aphasia or hemispatial neglect. Good comprehension/cognitive ability.	To examine the clinical effects of combining motor imagery-based neurofeedback training with bilateral repetitive transcranial magnetic stimulation for upper limb motor function.	Ten sessions of bilateral rTMS were administered alone (therapy A) or in combination with 12 non-consecutive sessions of motor imagery-based neurofeedback training (therapy B). Patients received therapies in sequence AB or BA, and were assessed before and after each therapy, and then after 15 days.	FMA-UL, Hand grip strength, NSA, 9-HPT, Computerized FTT, AMI.	Although both therapies showed significant results in proprioceptive awareness, sensory and kinaesthetic functions, the B therapy proved more effective regardless of whether it was performed in AB or BA mode.

Dynamic Gait Index (DGI), Timed Up and Go Test (TUG), Walking test (WT), Wisconsin Gate Scale (WGS), Walking While Talking (WWT), Barthel Index (BI), Berg Balance Scale (BBSI), Sit to Stand Test (SST), Rivermead Mobility Index (RMI), Mini Mental State Examination (MMSE), Timed Up & Go (TUG), Emory Functional Ambulation Profile (E-FAP), Medical Research Council (MRC), Biodex Balance System (BBSII), Isokinetic Dynamometer (BID), Tibialis Anterior Activation Index (TAAI), National Institute of Health Stroke Scale (NIHSS), Montreal Cognitive Assessment (MoCA), Mental Fatigue Scale (MFS), Modified Rankin Scale (mRS), Hospital Anxiety and Depression Scale (HAD), Euro-QoL-5 Dimension Questionnaire (EQ-5D), Stars Cancellation Test (SCT), Active Range of Motion (AROM), Letter Cancellation Test (LCT), Comb and Razor Test (CRT), Cervical Joint Position Error Test (CJPET), Numerical Rating Scale for pain (NRSP), Occupational Gaps Questionnaire (OGQ), Work Ability Index (WAI), Cognitive Rehabilitation Task Force (CRTF), Process-based method (APT), Activity-based method (ABAT), Sub-acute group (SAG), Post-acute group (PAG), Fugl-Meyer Upper Limb Assessment (FMA-UL), Nottingham Sensory Assessment (NSA), Nine-Hole Peg Test (9-HPT), Finger Tapping Task (FTT), Arm Motricity Index (AMI).

All the studies included in this review were written in English and published between 2015 and 2024. The sample size ranged from 12 to 101 patients between 30 and 83 years of age. All studies that were considered for this study had to fulfil specific inclusion and exclusion criteria. The total score obtained at NOS was 4 and 5 for two studies (46, 47), 6 for five studies (48–52), 7 for one study (53), and 8 for two studies (54, 55).

Concerning the type of design, seven studies were prospective (46, 48, 50, 51, 53–55) while three were retrospective (47, 49, 52). Among the prospective studies one was a pilot study (47), one was a semi-randomized controlled trial (49), and one was an exploratory crossover trial (52). On the other hand, retrospective studies included five randomized controlled trials (48, 50, 53–55), one non-randomized non-controlled study (46), one semi-randomized controlled trial (49), and an observational study (51).

All patients enrolled in these studies were diagnosed with stroke. Among the patients involved, four studies distinguished between ischemic and hemorrhagic stroke (46, 50, 52, 54), one enrolled only ischemic stroke patients (51), while the other five did not (47–49, 53, 55). One study makes the distinction between patients with stroke and with traumatic brain injury (55).

With respect to time elapsed from stroke event, five studies refer only to patients with chronic stroke (47–50, 53), two only treated patients with subacute stroke (51, 52), included both types of patients (54), and two made no reference to any such differences (46, 55).

Most studies report the site of the lesion (46–48, 51, 53, 55), while others do not (49, 50, 52, 54). The most frequently lesion site reported was the middle cerebral artery area (46, 48, 51, 53). The symptomatology of enrolled patients was specified in all but one study (54). Specifically, some studies refer to an upper (48, 52) or a lower (47) limb paresis/motor impairment. Other studies refer to patients with hemiparesis/hemiplegia or more generally deficits in mobility and balance (46, 51, 53). Interestingly, only two studies treat patients with attention disorders (55) or neglect (50). None of the articles examined utilized specific treatments aimed solely at recovering BA. Treatment types were diverse and ranged from tactile/auditory (47) or electrical/magnetic stimulation protocols (49, 52), to combination therapy/dual task protocols (48, 50, 51, 53). Two studies referred to the immersion of the rehabilitation process in a natural (54) or virtual environment (46).

The review focused on rehabilitation rather than assessment, as this seemed to be a topic less addressed in the literature. Tambone et al. (46) proposed a virtual reality training to stimulate active and repetitive motor practice of impaired body parts. The underlying idea is that the motor system may be activated by the body ownership namely the subjective experience of being the owner of one’s body without any actual motor execution. Humans’ perceptual status that the body belongs to the self is known to arise and be maintained from body-related afferences (i.e., visual, tactile, proprioceptive, and auditory signals) that constantly reach the physical body. On this line, Gomez-Andres et al. (47) in their work used feedback-based rehabilitation via a real-time movement sonification. During the walk, auditory feedback provided to the patient supplementary information about its body position, increasing movement, coordination and BA. Some studies such as those by Sengar et al. (48) and Battesha et al. (53) proposed training that considered both motor and cognitive aspects. In the former case patients received dual-task balance training with postural (step forward, backward and sideway) and cognitive tasks (remembering of words, counting forward and backward by adding 3 to the digits). On the other hand, patients in the work of Battesha et al. (53) received physical rehabilitation in addition to maze control training. In this paper patients were standing on a platform that had a motion, and they were asked to follow a set of targets through a maze. The participant moves the cursor to each target as it blinks and did not let the cursor touch a wall of the maze. Gandolla et al. (49) used functional electrical stimulation (FES) as adjunctive therapy in stroke patients. This treatment is primarily used for the orthotic correction of foot drops, but a proportion of patients relearn the ability to voluntarily dorsiflex the ankle without the device (“carryover effect”). They found that patients who experienced the carryover effect have responses in supplementary motor area and primary motor area that correspond to healthy controls, while these regions ‘activity is diminished in patients without the carryover effect. A study of Palsadottir et al. (54) assessed a nature-based rehabilitation able to provide multiple sensory stimuli environment and nature-based occupations in addition to standard care. The results of the study showed no significant differences between experimental and control groups, but patient compliance was high. Varalta et al. (50) in their study used elastic therapeutic taping to reduce neglect hemispatial symptoms. Patients were taped with elastic strips for a total of 30 days. Rotation, flexion and inclination was improved after the treatment, but there was no effect on visuo-spatial/motor abilities or BA. All the studies resulting from the review included a neurocognitive assessment, but none of the studies reviewed proposed a stimulation or rehabilitation pathway of cognitive abilities. In addition, treatment outcomes were considered only from a “physical” perspective (e.g., improvement in walking, balance, or movement of a certain body part) and not proprioceptive.

Chiaramonte et al. (51) in one of their group of patients they combined traditional rehabilitation exercises with goal-oriented proprioceptive exercises of increasing difficulty based on the patient’s characteristics and improvement. These exercises were aimed not only at improving motor performance, but also at improving the representation of the body and its movements in space.

In the study of Markovic et al. (55) a multidisciplinary team performed a rehabilitation treatment for both acute and subacute groups. The latter, in addition to regular rehabilitation, undergoes two types of training: attention process training (APT) or activity based attention training (ABAT). The first one is a neuropsychological structured process-oriented attention training who comprises repetitive exercises and meta-cognitive strategy training. It aimed to improve training tasks and global attention performances to allow better insight, motivation and generalization to daily life activities. On the other hand, ABAT is an occupational intervention that through attention-demanding everyday activities aimed at the training of functional skill (personal care, household activities, work, leisure, social activities).

Sanchez-Cuesta et al. (52) performed bilateral rTMS sessions in combination with motor imagery neurofeedback training. During this training patients were asked to perform motor actions such as they would do in real life while they had head-mounted virtual reality (VR) headset with a 90° horizontal field of view, and haptic feedback delivered via 2 controllers in both hands.

## Discussion

The management and rehabilitation of complex patients such as those with stroke constitutes a difficult challenge. Depending on the type of injury, rehabilitation can require a considerable amount of time (56). Moreover constant/fluctuating alterations in the patients’ neurocognitive performances, make any rehabilitation treatment decidedly difficult. Since BA in these patients is often impaired and its recovery does not always occur spontaneously but has to be elicited and sustained by neurorehabilitation (57, 58) it is imperative to deepen knowledge about the approaches that are most widely used in literature and their effects. Therefore, in this study we aimed to assess whether specific BA treatments are used in recent literature and/or whether BA is considered as an outcome of body neurorehabilitation processes in stroke patients. To fulfil our purpose, 10 eligible studies were included in this review and were accurately examined. As shown in Table 1, the values for study quality measured by NOS ranged from 4 to 8, with an average of 6.2. Therefore, the overall quality of studies was found to be satisfactory, but the research design and type of treatment was varied. Indeed, results obtained showed that in general all articles delve into different types of treatment aimed at improving complementary aspects of BA (attention, walking, balance, awareness about positioning and movement of specific body parts) but without ever going into specific BA focus. This could have two possible explanations. First, although the results of our work show that not all studies report this information exhaustively. This could be attributed to a lack of specific assessments for body schema disorders and to the choice of treatment that depends on the extent, location and time elapsed since patient’s injury. Indeed, in stroke patients motor and BA recovery is facilitated and modulated by neural plasticity, that is enhanced early after stroke (59). Motor recovery would appear to occur mainly within a time frame of between 3 and 6 months, and after this critical period it may further improve, worsen or remain stable (60, 61). The studies considered in this review included patients with subacute or chronic stroke. In the subacute phase (1 week to 3 months after stroke) there is a reorganization of neural networks that is accompanied by the recruitment of areas attached to the lesion to compensate for the motor and cognitive functions that have been lost (62, 63) recovery becomes slower and less spontaneous, requiring more intensive and prolonged rehabilitation stimuli (64). On the other hand, it may be difficult to find patients with pure deficits in BA also because it is a complex concept that can be indirectly measured by different measures: proprioception, interoception, body ownership, body parts position, attention, description of mental image of body parts, awareness, balance, walking, etc. (65). This makes it equally difficult to isolate one element and estimate what impact it has on the recovery or quality of life of these patients. Elements that are present in stroke patients are various and are often closely related. In this regard Serrada et al. in one of their studies (66), assess BA using both the Body Perception Disturbance (BPD) and the MAIA. BPD is an instrument structured in three sections (body perception self-assessment, body distortion questions, level of concern about weight and shape) used to identify patient disturbances in body perception (67, 68). This scale can be administered to different age groups and both sexes and can be used with different clinical populations that range from stroke to eating disorders or chronic pain patients (69). The achievable score ranges from 0 to 57, with higher scores that represent greater body perception disturbance.

On the other hand, as we mentioned the MAIA scale is a tool designed to evaluate the capacity to perceive, respond and interpret internal body sensations. This instrument consists of several subscales, each measuring a specific aspect of interoceptive awareness (noticing, distracting, worrying, attention regulation, emotional awareness, self-regulation, body listening and trusting). Despite its multidimensionality, this instrument has the limitation of not being suitable for administration in the acute phase. The authors themselves (70) not only propose assessment tools, but also refer to specific training for the rehabilitation of BA. The Feldenkrais Method (FM) is an approach that aims to help individuals self-organize and become self-directed learners by enhancing their consciousness and awareness of how their bodies perform tasks (71). This approach utilizes perception and imagination to improve coordination, control, movement ease, efficiency, effectiveness, and overall function (72). Specifically, changes in motor behavior occur through the practice of cognitive inquiry, which involves shifting the focus from the goal of movement to bodily sensations and perceptions experienced during specific guided movements (73). The theoretical framework is built upon intrinsic and extrinsic feedback, task-specific and variable practice, repetition, part and whole practice, transfer of learning, mental practice, exploratory learning, and body awareness/image (74, 75). Indeed, it is believed that the formation of new internal representations in the brain leading to more efficient motor planning and execution. Although some reviews in the literature agree that the number and type of studies and patients on whom FM is performed is too diverse, the same works recognize its potential in improving body awareness (especially with respect to attention, dexterity, and coordination) (76, 77). It is no coincidence that many of the approaches used by the studies involved in the review draw on these constructs. Motor and cognitive aspects were considered by Sengar et al. (48) and Battesha et al. (53). In both studies patients received composite training with motor (postural/physical rehabilitation) and cognitive tasks (memory/attentive − executive tasks). According to the task-integration hypotesis the complementary or concurrent performance of two tasks allows patients to develop and improve co-ordination skills (78). Indeed, in Sengar et al. (48), the simultaneous performance of motor and cognitive tasks optimized the interaction between the vestibular, visual, and somatosensory systems producing an improvement on BA that resulted in greater motor control, balance, and attentional resources. In Battesha et al. (53) instead, the implementation of a cognitive maze control task to the conventional physical therapy rehabilitation enhanced even the peripheral somatosensory stimulation, inducing an improvement on proprioception, kinesthetic awareness, motor/posture control and reducing risk of fall. Feedback construct was deployed by Gomez-Andres et al. (47) that used real-time movement sonification feedback. The continuous emission of feedback and the rhythmic nature of the sound allows the patient to synchronize with it, gradually becoming more conscious of its body movement. Through this method, individuals learn to refine their movements and improve their functional capabilities by engaging in a deeper awareness and understanding of their bodily actions, space position and coordination (79). Moreover, sonification approaches usually transform kinematic and dynamic movement through distinct sound components (e.g., pitch, loudness, rhythm, and timbre) making the stimulation multimodal and improving the sensorimotor representation of the movement (80, 81). A rich neuroanatomical interconnectivity was found between several distant cortical and subcortical brain areas, including premotor cortex, supplementary motor area (SMA), pre-SMA, basal ganglia, thalamus, cerebellum, supplementary motor area (SMA) and pre-SMA, premotor cortex, and the auditory cortex (82, 83).

Pálsdóttir et al. (54) in their study used a nature-based rehabilitation setting to provide multiple sensory stimuli environment and nature-based occupations in addition to standard care. The environment, setting and the manner in which the process takes place turn out to be key aspects in the conduct of neurorehabilitation interventions. A recent review of Vibholom et al. (84) showed that nature-based rehabilitation has proven effective in improving motor and cognitive functions and quality of life. In addition to patients, nature-based environment would also seem to be an advantage for healthcare workers, who perceive the workplace as more comfortable and less stressful (85). These data make it possible to hypothesise that in addition to the rehabilitation process, an important role is played by the hospital setting and the individuals involved in it, regardless of whether they are health workers or patients. In this respect, Awareness Through Movement (ATM), is configured as a community-based intervention which takes the form of a declination of the FM. In ATM a teacher guides structured sessions using verbal cues promoting engagement, adherence, quality, and feedback, and is accessible to individuals of all levels of physical fitness (86). At the beginning of the ATM session, patients are asked to scan their own bodies, allowing participants to pay attention to their current level of BA and identifying particular states and traits (87). The subsequent step consists in structured explorations of movement sequences with key prompts for attention, specifically focusing on noticing where movement does and does not occur in various body areas, as well as the level of ease or effort involved (88). The final stage of the approach is a review of the body scan to highlight any changes and learning that have occurred. However, experts must deliver at a central location and participants need to travel. Alternatively, home-based ATM classes are potentially more feasible and low cost, but engagement and quality may be reduced (89). Although the work in the literature is generally positive about these approaches, the results obtained often consider too diverse outcomes, and consequently they are controversial (90). Lindvall et al. (91) described Body Awareness Therapy (BAT), a physiotherapy group technique that focus attention on the action and experience of movement and that is perceived positively by patients, who see it as something stimulating and able to bring them improvements (92, 93). However, in one of their previous studies, they found no differences between control and experimental group treated with a 8-week BAT program (94). On the contrary Bang et al. (95, 96) found significant improvements treating experimental group with BAT 3–4 weeks BAT program.

Like BAT, even the other treatments described above allow patients to confront their limits, motivate them to improve, and allow the development of an integrated knowledge of the body and its movements. However, studies results, interventions typologies, and the way they are applied still vary too much between the different studies in the literature, and for this reason the topic of BA should be further investigated. Among the strengths of this study is certainly the choice of the topic of BA, which allowed comparison and updating of different studies and rehabilitation approaches. A complete and effective rehabilitation treatment cannot disregard the cognitive, psychological and relational aspects of the patient. The fact that one or more of these aspects is most often neglected undoubtedly constitutes an element that needs to be addressed and improved (97).

This study is not without limitations. Although an attempt was made to do a comprehensive job by including a large number of articles, the databases selection may have left out some data from studies in other databases. In the same vein, the exclusion criteria adopted may have resulted in important information being overlooked, such as that derived from case reports.

## Conclusion

Our work showed that in general all articles delve into different types of treatment aimed at improving complementary aspects of BA, but without ever going into specific BA focus. Moreover, NOS assessment showed that the topic could positively benefit from future randomized controlled trials in larger samples. None of the studies discussed investigates a specific treatment for the rehabilitation of BA. They all use a holistic approach that sees BA as a part of a broader construct. While this can be advantageous because it allows for the treatment of multiple aspects of motor coordination and human movement, it risks being less precise and less specific than rehabilitation of a specific deficit. A precise definition of the BA deficit makes it possible to identify its dysfunctional components (e.g., proprioception, interoception) and to provide physicians and health professionals with fundamental information for the patient’s rehabilitation pathway. Therefore, it would be advisable to standardize *ad hoc* batteries or tests that allow the identification, definition and evaluation of the type of BA deficit and its extent. Consequently, the planning of rehabilitation programs could be aimed at recovering dysfunctional components.
